# Testamentary capacity assessment in dementia using artificial intelligence: prospects and challenges

**DOI:** 10.3389/fpsyt.2023.1137792

**Published:** 2023-05-31

**Authors:** Alexandra Economou, John Kontos

**Affiliations:** ^1^Department of Psychology, School of Philosophy, National and Kapodistrian University of Athens, Athens, Greece; ^2^Department of History and Philosophy of Science, National and Kapodistrian University of Athens, Athens, Greece

**Keywords:** testamentary capacity, capacity assessment, dementia, cognitive impairment, explainable artificial intelligence, decision support systems

## Abstract

Testamentary capacity (TC), a set of capacities involved in making a valid Will, has become prominent in capacity evaluations due to the demographic increase in older persons and associated increase in cognitive impairment. The assessment of contemporaneous TC follows the criteria derived from the Banks v Goodfellow case, which do not bind capacity solely on the basis of presence of a cognitive disorder. Although effort is being made for establishing more objective criteria for TC judgment, variations in situational complexity call for incorporating the different circumstances of the testator in capacity assessment. Artificial intelligence (AI) technologies such as statistical machine learning have been used in forensic psychiatry mainly for the prediction of aggressive behavior and recidivism but little has been done in the area of capacity assessment. However, the statistical machine learning model responses are difficult to interpret and explain, which presents problems with regard to the new General Data Protection Regulation (GDPR) of the European Union. In this Perspective we present a framework for an AI decision support tool for TC assessment. The framework is based on AI decision support and explainable AI (XAI) technology.

## Introduction

Capacity is a legal and clinical term that refers to the ability to perform certain functions or to make autonomous decisions. It is a time-limited and task-specific ability that a person is presumed to have unless proven otherwise (1). Testamentary capacity (TC), a specific civil competency, describes a complex set of capacities involving the decision to make a valid Will. Like all mental capacities, it is based on the understanding of relevant facts and information, the appreciation of the nature of the situation and the reasonably foreseeable consequences of such a decision (2).

TC has become the focus of capacity evaluation in recent years due to the demographic increase in older persons, with the concomitant increase in dementia and cognitive impairment (3), as well as societal changes involving the rise in different forms of family unions, divorce, and remarriage rates (2).

## Criteria for testamentary capacity

The ascertainment of contemporaneous TC requires understanding of (a) the nature of the act of making a Will and its consequences; (b) the extent of one’s assets; (c) the claims of those who might benefit from the will; (d) the impact of the distribution of the assets; and additionally, (e) that the testator is free of mental disorders or delusions that influence the disposition of assets (4). These criteria, derived from the seminal Banks v Goodfellow case on TC in England in 1870, were ground-breaking because of the notion that capacity is not bound by a diagnosis; the real question according to the Chief Justice was whether the testator was of sound mind to make a Will and not whether he suffered from “general insanity.” Therefore, the presence of a cognitive disorder does not in itself preclude capacity although it may support the clinical assessment. Changes in the environmental context of TC since that time have led to a proposal for an updated test for TC that explicitly incorporates situational complexity, as well as greater clarity regarding the cognitive underpinnings of “understanding” (5).

Whereas the Banks v Goodfellow case was the result of a case of psychosis, dementia is far more prevalent in recent times (5). The challenge in assessing TC in dementia is its progressive nature impacting on cognitive ability and the fact that different types of dementia are characterized by differing profiles of cognitive loss. In accordance with the Banks v Goodfellow case, a diagnosis of dementia does not automatically necessitate loss of TC. The question that therefore arises is what cognitive abilities are required to satisfy the criteria for establishing TC in dementia (3).

## The assessment of testamentary capacity

Analysis of TC in legal cases can be either contemporaneous with a Will execution (the main focus of this paper) or retrospective. Undue influence, a conceptually separate but related to TC issue refers to wrongful persuasion that overpowers the will of a person and induces the person under the will of another. It includes the criterion of the testator’s susceptibility due to a psychiatric or medical condition, which renders the testator vulnerable to the influence of others and can invalidate a Will (6, 7).

The assessment of TC may be informed by expert medical or psychological assessment. The expert selected must have the appropriate knowledge and skill to provide *an evidence-based assessment and the reasoning for their opinion* with the appropriate legal framework (8). Resulting clinical classification of TC can be high, low, or borderline (6) but ultimately it is the court that makes the final decision (9). Because TC varies as a function of complexity within a testator’s environment and of the Will itself, a certain level of cognitive impairment may not interfere with TC in one individual with few assets in a simple context but may do so in a more complex or conflictual context (4, 5).

### Forensic assessment instruments

TC has been typically assessed by semi-structured interviews, such as the Contemporaneous Assessment Instrument (CAI), which follows the six criteria established by Applebaum and Grisso that were pertinent to the four legal requirements for decision-making (understanding the nature of the act and its effect; the extent of the property the testator is disposing; the claims which the testator should give effect; to be free of a disorder of mind preventing the disposing of one’s property). These are that the functions assessed should be conceptually close to the standards of competence; the content of the instrument should be pertinent to the distribution of one’s estate; the instrument should be meaningful to the person assessed; the instrument should be sufficiently standardized to enable comparisons within and across research groups; the measures must have objective and reliable scoring criteria; and the instrument should be practical for use. By definition, some of the criteria must be specific to the person being assessed (2).

The Testament Definition Scale (TDS) is a short rating scale designed to evaluate and quantify the individual’s ability to give an exhaustive definition of what a testament is, thus addressing the first criterion derived from the Banks v Goodfellow case (10).

The Legal Capacity Questionnaire (LCQ), a screening instrument for attorneys, examines three of the four legal requirements of TC using true/false, multiple-choice, and open-ended questions. On the basis of limited normative data for older adults and correlational analyses with mental status and behavioral instruments, cut scores have been created for high, borderline, and low capacity. The LCQ is geared toward older clients with cognitive disabilities, is administered in a standardized manner and is norm-referenced but lacks reliability and validity information, the criterion of understanding what a Will is, and the investigation of psychotic symptomatology and undue influence (6).

The Testamentary Capacity Assessment Tool (TCAT) (11) is a brief instrument that has been validated in older adults with dementia by comparing the scores with the opinion of a forensic psychiatrist regarding the TC of the patient, and the judgment of a senior Judge. It has been standardized in an Italian neurologically healthy sample (12). The TCAT examines orientation, autobiographical memory, knowledge of financial parameters, intention and judgment. Behavior pertaining to presence of acute psychopathology is also documented. The instrument can be used to screen the clearly competent testator or as corroborative evidence of an expert’s judgment. Because it is based only on the interview with the patient independent of collateral information it is impartial to potential conflict within the patient’s family.

The more recent Testamentary Capacity Instrument (TCI), Martin et al. (13) is an interview-based instrument that assesses the aforementioned four criteria of TC in a standardized interview-based format, utilizing a variety of question formats depending on the section and provides objective scoring criteria. Preliminary data showed that it was a valid measure of TC with good inter-rater reliability and internal reliability for the criteria for which such measures could be calculated. No cut scores were proposed for determining capacity, because the TCI was intended to serve as a source of additional information for making clinical judgment.

### Neuropsychological assessment

Neuropsychological assessment is not a source of direct evidence for TC, although it can contribute to the clinical and diagnostic context for its assessment (14) and can provide important evidence where incapacity is alleged to invalidate a Will (3).

Cognitive screening measures such as the MMSE are not reliable and valid measures of TC and are not sensitive to impairments affecting judgment and reasoning despite their wide use in capacity evaluations. The cognitive domains of complex attention, executive function, learning and memory, language, and social cognition may affect TC in different ways, although there is limited information on how impairments in these domains impact on the Banks v Goodfellow criteria (3). A recent study that correlated performance on a neuropsychological battery to the TCI in a small number of cognitively healthy persons and persons with AD showed that measures of executive function, verbal memory, and semantic knowledge accounted for over 70% of shared variance with TCI performance and each measure correlated with each of the four TCI elements, suggesting a similar multidimensional cognitive basis for each TCI component (15).

### Retrospective assessment

Although a contemporaneous assessment is desirable, many situations require a retrospective assessment of TC. A proposed methodology along with pitfalls of conducting a retrospective neuropsychological assessment are described in Zago and Bolognini (16). Of interest is the inclusion of handwriting analysis, evaluated longitudinally, as a complementary tool for the evaluation of cognitive status, both with respects to formal aspects of the writing skills (spelling, grammar, layout) and logical consistency of the written text. The specifics of conducting such an analysis and deriving a “writing score” in the posthumous challenge of a will are provided in Balestrino et al. (17). Preliminary evidence points to the association of a low writing score with abnormal performance on mental status tests but the establishment of the validity of the method requires further research (16, 17). An assessment of medical history should precede such assessment to exclude testators with neurological conditions impacting on graphomotor skills, such as Amyotrophic Lateral Sclerosis or Parkinson’s disease, or other medical conditions such as rheumatoid arthritis.

### Challenges in testamentary capacity assessment

Providing evidence-based assessment and reasoning for a TC opinion requires training that the person conducting the assessment may not have. Moreover, while effort is being made for establishing more objective criteria for TC judgment, assessment also needs to be tailored to the individual situation of the testator.

Evaluation of TC in aphasia and in neurodegenerative disorders characterized by progressive language impairment are a challenge to TC evaluation, as the testator’s ability to verbally express the components of TC and explain decisions is essential for establishing TC [e.g., case by (18)]. The assessment of TC in persons with severe aphasia is therefore problematic or even impossible, and techniques and formats that may facilitate the evaluation, such as prompted recall or multiple-choice formats for recognition memory, are not without drawbacks (13). It is proposed that persons with aphasia should have greater access to the courts through the facilitative support of speech-language pathologists (19, 20), which is in line with the supported decision-making trend of disability rights law (21).

In cases of severe aphasia, nonverbal neuropsychological assessment could serve as a proxy measure although it cannot replace the TC criteria. Linguistic deficits notwithstanding, other cognitive functions may be affected in aphasia (22), which may be independently impaired and may impact on mental capacity. To the best of our knowledge, there are no studies on the association of nonverbal assessment with neuropsychological tests of cognitive function that can be used in TC assessment. The establishment of such an association would facilitate the clinical and diagnostic context for TC assessment in aphasia and some types of dementia.

## Forensic psychiatry and artificial intelligence

Artificial intelligence (AI) technologies and applications are ubiquitous but are not a monolithic technology. AI technologies can involve rule bases, language models, and machine learning (ML); applications can be trained using supervised ML and reinforcement learning or can look for patterns using unsupervised ML (23). ML programs are generally not designed to offer an explanation for their predictions, and different ML algorithms vary in degree of transparency in decision-making. Therefore, when a model fails to make a prediction in a different sample it is difficult to analyze the reason (24).

The computer generation of explanations dates back to the 80s with the implementation of the MYCIN expert system based on the tracing of rule-based reasoning for treatment advice for patient symptoms (25). More recently, a system was presented that generated explanations based on the tracing of text-based reasoning (26). Recent initiatives like the DARPA explainable artificial intelligence (XAI) program (27) have brought explainability back into focus (28), although the basic aspect of XAI by means of knowledge representations of a set of concepts within a domain, and statistical, formal or causal models remains mostly unexplored. The source of this neglect is the extreme popularity of statistical methods that generate “black boxes,” which do not lend themselves to human understanding of their operations. Such models are constructed by “blindly” fitting parametric functions to the data in a curve-fitting process by automatically adjusting the values of thousands or even millions of parameters that have no meaning and no causal relations to the problems under study (29).

The role of AI in forensic psychiatry has received increased attention in recent years, with the majority of studies coming from the area of predicting violence risk in patients in psychiatric institutions or in people held in custody, e.g., [(30–34); review by (35)], and a review on the controversial topic of use of brain reading AI for neuroprediction of violence by Tortora et al. (36), and the prediction of future offenses and dangerousness in persons with psychiatric disorders, e.g., [(37, 38); review by (39)]. The advantage of ML is that it can combine large numbers of data and investigate large numbers of predictors in nonlinear and interactive ways. ML models are particularly useful for criterion-referenced assessment, designed to predict specific behaviors and outcomes, rather than for norm-referenced assessment, designed to measure constructs such as personality and mental status (24). Not surprisingly, most studies in forensic psychiatry involve criterion-referenced prediction rules.

### Ethical and legal issues in the use of AI

The new General Data Protection Regulation (GDPR) of the European Union enacted in 2018 prohibits automated decision-making, that is, making a decision solely by automated means without any human involvement, if a decision produces legal or similar consequences (23). The GDPR has also created a “right to explanation” of how an AI system works and how it reached a decision, which is especially pertinent for ML models that are inherently challenging to interpret (23, 40) and raise ethical issues, particularly in legal settings (24). It has been argued that the explanations that derive from correlational computations can lead to spurious correlations rather than cause-effect relationships, resulting in erroneous or biased explanations (41). International guidelines for trustworthy AI place explainability in the center of trustworthy systems and regulatory guidelines (42, 43). Therefore, there is a growing need for the design and development of transparent and interpretable AI models and explanations that reflect the needs of the users (44, 45).

### Testamentary capacity and artificial intelligence

Little attention has been paid to the potential contribution of AI to capacity assessment, although a theoretical paper has been written on the broader civil competency of financial capacity in cognitive disorders that includes AI (46). According to one perspective, capacity assessment does not lend itself to formalization; in mental health the notion of incapacity is a complex legal, clinical, ethical, and social construct. Testing for it varies according to the specific circumstance for making a decision, making it difficult to formalize mathematically as required by AI (47).

On the other hand, the use of ontologies for enhancing human understandability of global post-hoc explanations of black-box models, as presented in Confalonieri et al. (48) may be developed for machine analysis of human explanations. Using this reverse reasoning, it is the *human explanations* regarding the evaluation of TC that could be subjected to machine analysis. Thus, instead of humans evaluating machine-generated explanations as to their correctness, AI would be called upon for the evaluation of human-generated explanations of a person’s behavior, to facilitate the decision of the expert. The decision of the AI system can be explained using XAI methods in order to increase the trust of the human evaluator in it. The explanation that a person provides for a certain behavior or decision could be processed by a natural language programming (NLP) system in order to draw conclusions for the assessment of one’s TC.

Using NLP methods proposed for assessing artificial agents’ responses in similar situations (49), “what” questions may be used for the understanding of the nature of the act of making a Will and its consequences as well as knowledge of the extent of one’s assets, and “why” questions may help disclose the testator’s understanding of the claims of those who might benefit from the Will and the impact of the distribution of the testator’s assets. The computer analysis of the human explanations mentioned above may contribute to offering advice to the expert making the assessment as to their correctness. This analysis could be supported by an explanatory model of the kind proposed in Pfeiff (50) of explanatory practices in psychotherapy intended to make the patients assume responsibility for their symptoms. Two different notions of rationality are used: theoretical rationality, the adoption of beliefs that cohere with one’s relevant background knowledge; and pragmatic rationality, the adoption of beliefs that are consistent with one’s goals. The notion of dysfunctionality is proposed in which a decision is dysfunctional if it does not satisfy one’s needs and may even cause harm. By analogy, the notions could be adapted to the assessment of TC.

For a retrospective analysis of handwritten or written texts AI offers some tools for their automatic analysis that takes into account both the formal aspects of the writing skills (lexical, syntactic, semantic and discourse level) and the spatial orientation in cases of handwritten texts. There is a large body of research that presents methods for evaluating these aspects of natural language in texts using AI [e.g., (51, 52)]. The identification of emotional state through the analysis of handwriting features is a new area in AI (53) that may be extended to the identification of handwriting features that reflect cognitive state.

To develop and test such an XAI based decision support tool would require a corpus or dataset of explanations provided by persons of varying TC, together with expert assessment of the state of their TC. However, the presence of a language disorder would invalidate the method proposed for assessing reasoning capacity because an AI system analysis of the verbal explanations of the decisions of the testator presupposes lack of language disorder of the testator. It is almost impossible to construct an AI system assessing human explanations that do not exhibit correctness in all the linguistic aspects to be tested, as listed in Table 1. Therefore, a challenge for the NLP system supporting the experts in their assessment of explanations would be to detect a language disorder as distinct from problems with reasoning and filter out the aberrant explanations before they are submitted to the XAI decision support system (DSS). Table 1 lists the basic tests to be performed by the NLP system during the filtering process.

**Table 1:** Human explanations as a tool for TC assessment.

NLP analysis of human explanations
Lexical correctness, i.e., correct choice of words
Ontological correctness, i.e., correct grouping of objects, events, and concepts
Syntactic correctness of the sentences
Selection restrictions correctness, i.e., correct choice of verb arguments
Semantic correctness of the sentences, i.e., constructing sentences with semantically consistent combination of words
Logical correctness of conclusions drawn from the facts
Decision correctness, e.g., decisions consistent with the relation of the beneficiaries to the testator

### Objectives of an XAI system for TC assessment

The design and implementation of an XAI DSS for TC assessment would have the following characteristics: (a) be a truly mixed human-AI system (b) be trustworthy, (c) utilize tacit knowledge of area ontology and simulation models, (d) be tested for its adaptation to the users regardless of their age, race, gender, or capabilities, and e) adapt its explainable decision support and explainable question answering “behavior” to the needs of the users. A mostly unexplored area is the variation of the explanation generation of the testator spanning several axes, such as from special to general ontology levels. This variation of the style of explanations would depend on the educational level and background of the testator. The explanation analysis software must be capable of processing a variety of styles of explanations, which is an open research question (see Appendix in Supplementary material for some possible variations to guide XAI experts).

Area under the curve (AUC) could be used as a test statistic to describe the ability of the tool to discriminate between capacity and incapacity and to compare the performance of different tools and methods, after Garb and Wood (24).

## Conclusion

The concepts proposed in this paper are preliminary and aim to address some of the challenges of TC evaluation. An AI decision support tool for TC assessment could be a hybrid system based on expert knowledge (rule-based) using causal relationships as well as data-driven knowledge (statistical ML). Such a system would support rather than replace the clinical decision of the expert, which can be high, low or borderline TC depending on the integration of evidence. An explainable, question answering DSS is proposed for the evaluation of the reasoning of the expert making a TC assessment. The XAI system would use natural language and would be developed and tested on a corpus or dataset of explanations provided by persons of varying TC. Such a system would be adaptable to the circumstances of the testator and would utilize tacit knowledge for human empowerment.

## Data availability statement

The original contributions presented in the study are included in the article/Supplementary material, further inquiries can be directed to the corresponding author.

## Author contributions

AE conceptualized the general idea and drafted the manuscript. AE and JK conceptualized and wrote the specific ideas presented. All authors contributed to the article and approved the submitted version.

## Funding

Open access funds were provided by the Special Account for Research Grants of the National and Kapodistrian University of Athens.

## Conflict of interest

The authors declare that the research was conducted in the absence of any commercial or financial relationships that could be construed as a potential conflict of interest.

## Publisher’s note

All claims expressed in this article are solely those of the authors and do not necessarily represent those of their affiliated organizations, or those of the publisher, the editors and the reviewers. Any product that may be evaluated in this article, or claim that may be made by its manufacturer, is not guaranteed or endorsed by the publisher.
