# Supplementary material for: Testamentary capacity assessment in dementia using artificial intelligence: prospects and challenges
Source: Front Psychiatry. 2023 May 31;14:1137792. doi: 10.3389/fpsyt.2023.1137792 (PMC10264688; doi:10.3389/fpsyt.2023.1137792)
Supplement: Supplementary file 1 [file Data_Sheet_1.PDF]

Appendix. *Possible variation axes for the testator explanation analysis system to guide the XAI expert*

**Explanation variation axis**	**Range**
1. From verbose to concise text	From long and verbose (which is easiest to generate automatically) to concise
2. From a formal to a qualitative model	
3. From statistical to causal model learning	
4. From purely textual to purely pictorial	Variable degrees of reliance on textual or pictorial representation of explanations
5. From oral to visual	Explanations that users prefer to hear or see
6. From a term definition to documentation of publications	The user may ask for additional clarifications, ranging from the definition of an unknown term to supporting publications relevant to the issue
7. From factoid to deductive XQA	The questions of users to the XQA subsystem may be factoid, deductive or both
8. From colloquial to formal language	The variation of the language used to express the explanation can range from colloquial to formal, such as use of scientific terms or jargon
